# The effects of a four-month skateboarding intervention on motor, cognitive, and symptom levels in children with ADHD

**DOI:** 10.3389/fped.2024.1452851

**Published:** 2024-12-02

**Authors:** Tabea Christ, Kim Joris Boström, Patricia Ohrmann, Henrike Britz, Heiko Wagner, Christiane Bohn

**Affiliations:** ^1^ Department of Movement Science, University of Münster, Münster, Germany; ^2^ Medical Directorate, LWL-Klinik Münster, Münster, Germany

**Keywords:** ADHD, children, skateboarding, movement therapy, motor ability, cognition

## Abstract

**Objectives and methods:**

This study aimed to investigate whether a four-month skateboarding workshop can positively affect attention-focusing skills and postural control in terms of static and dynamic balance in addition to symptoms of ADHD in school-aged children (*N* = 58). Kinematic and kinetic movement analysis, attention-focusing tests as well as symptom questionnaires were employed to measure differences caused by the skateboarding intervention. A weekly skateboarding workshop was conducted with children diagnosed with ADHD which intended to encourage children to autonomously engage in physical activity. Group differences were analyzed using a generalized linear mixed model. A partial correlation was used to investigate possible relationships between the variables.

**Results:**

A preceding analysis confirmed that children with ADHD perform statistically significantly inferior in the employed tests of balance and concentration ability than unaffected peers of the same age. The main analysis showed that after the skateboarding intervention, children with ADHD were able to improve cognitive and motor test performances as well as symptom expression significantly. Significant improvements were likewise found in the waitlist control group, these were however less pronounced compared to those of the skateboarding intervention group. The correlation analysis revealed that there is no reciprocal influence between cognitive and motor skills, nor between motor skills and ADHD symptomatology in the present patient group. Possible explanations are discussed.

**Conclusion:**

Skateboarding as a form of movement intervention can be considered an effective method for children with ADHD to deal with their symptoms and deficits. An intervention period of four months has helped children with ADHD discover and embrace an informal sport like skateboarding, thereby finding enjoyment in movement and practice a skill from intrinsic motivation. To retain the benefits, it is advisable to practice a sport on a long-term basis. Thorough conceptualizations to implement this type of therapy await further research.

## 1 Introduction

Attention deficit hyperactivity disorder (ADHD) is one of the most prevalent neurodevelopmental childhood disorders worldwide (1). Symptoms such as hyperactivity, impulsiveness, and inattention typically appear at pre-school age and may persist into adulthood, leading to lifelong difficulties in social functioning (1). ADHD is believed to be caused not only by a disturbance in the dopaminergic system but also by genetic predisposition and exposure to environmental risk factors, especially during the prenatal stage (2, 3). It can be classified as an externalizing disorder (4) due to the explicitness of its symptoms, which makes patients more likely to be noticed by their environment and receive a diagnosis.

Children with ADHD have a delay in motor development of almost two years compared to their typically developing peers (5). Tseng and colleagues (6) examined the relationship between motor performance and the central ADHD symptoms inattention, impulsiveness, and hyperactivity, finding that attention and impulse control were strong predictors of gross and fine motor skills in children with ADHD. Additionally, when using the Movement Assessment Battery for Children (MABC), children with ADHD demonstrated significantly poorer movement abilities compared to their neurotypical counterparts (7). Likewise, ADHD children exhibited hyperkinesis, poor timing and force control, poor balancing, difficulties in learning and performing a variety of motor skills, as well as significantly inferior fine motor skills tested with a Purdue Pegboard (7).

Mao and colleagues (8) specifically investigated the static and dynamic balance abilities in children with and without ADHD. Their findings confirmed that children with ADHD perform inferior in the balance subtests of the MABC and the Bruininks-Oseretsky Test of Motor Proficiency (BOTMP) to unaffected controls. Previous research has shown that ADHD patients have a smaller cerebellar volume (9, 10), and since the cerebellum plays an important role in postural control and motor coordination, balance dysfunctions are not surprising (8).

Although considered a spectrum disorder with various degrees of symptom severity (11) children with ADHD commonly exhibit deficits in several cognitive domains, including inhibition, working memory, temporal discounting, decision-making, timing, and reaction time variability (12). Deficits in inhibition and working memory have been reported to affect only a minority of children with ADHD (13, 14). The evidence for impaired motor inhibition is much stronger than for cognitive inhibition (12). Timing deficiencies seem to play a significant role in ADHD-related problems, primarily affecting temporal information processing and motor timing (12). Individuals with ADHD may experience time passing more slowly, which could partially explain their difficulty in waiting and the resulting impulsivity (12). Response time variability (RTV), being a non-executive component of cognitive functioning is a common attribute associated with ADHD (12). Studies on RTV, specifically error measurement and reaction time experiments, have consistently distinguished between ADHD patients and neurotypical controls (15, 16). Additionally, RTV is believed to contribute to abnormalities in attention and behavior (16).

Although ADHD is not curable, two types of symptom treatment have shown great success (17). For patients under the age of six years, the American Academy of Pediatrics (AAP) recommends behavior therapy for the child in addition to parental training (18). Behavior therapy includes a learning theory known as classical conditioning, whereby the child learns to enhance positive behaviors and decrease problematic behaviors. Behavioral management should not be limited to the home environment with parents but may also be extended to classroom interventions involving teachers and peers (18).

For children above the age of six, a combination of behavior therapy and medication is recommended. Methylphenidate, a stimulant, has proven to be highly successful due to its positive effect on reducing the core symptoms of hyperactivity, impulsivity, and inattention (19). However, many adverse events have been reported in children and adolescents who have taken methylphenidate, often leading to withdrawal of the medication (19). Therefore, other treatment forms, such as movement therapy remain highly relevant as they are an essential addition to medication-based treatment.

Only a few studies have examined the effect of physical activity and movement on ADHD symptoms, and cognitive and motor performance of ADHD children (20, 21). Literature indicates a positive relationship between various forms of physical activity and cognitive functioning in children with ADHD (20). The authors investigated the impact of long-term physical activity on cognitive skills and motor performance in children with ADHD. They found that those who participated in a 12-week training program significantly increased their working memory and motor coordination compared to a wait-list control group (20). Likewise, Verret and colleagues (21) demonstrated that a ten-week fitness program improved information processing, sustained attention, motor skills, and behavior in children with ADHD.

Another therapy approach to treat ADHD is dance movement therapy (DMT) (22–25). DMT has shown to promote joy of movement and creative play with peers as well as to help individuals emphasize their strengths and improve their self-esteem (22). Improved ADHD assessment scores of teachers and parents further supported the effectiveness of DTM (25). Moreover, martial arts and mindful movement therapy have frequently proven to help ADHD-affected children deal with certain symptoms (26–28). Studies have demonstrated that participation in a motor program, in this case a trampoline training program, can have a significant impact on social participation in children with intellectual disabilities (29) and those with autism and/or sensory processing challenges (30). The authors concluded that an interesting and enjoyable intervention program is particularly useful for the given subject group to encourage an active lifestyle (29).

Several studies have demonstrated that a psychomotor intervention program can effectively help children counteract the impairments and symptoms of ADHD (31–34). Psychomotor activity strengthens the interconnection between bodily motor processes and psychological perceptual processes (35). This therapeutic approach mainly aims to enhance the patients' self-concept and self-efficacy by promoting their social-emotional development (35).

Although numerous different movement programs have been conducted with children diagnosed with ADHD, no research has yet examined the effect of a long-term skateboarding program on symptoms of ADHD as well as on motor and cognitive abilities. Casey and colleagues (36) established the benefits of therapeutic ice skating for children with autism spectrum disorder. Moreover, psychomotor therapy efficiently employs the roller board as sports equipment, teaching children to maintain balance on an unstable surface and improve social behavior. Our study is the first to allow conclusions about the effectiveness of a subcultural sport like skateboarding to reduce children's ADHD symptoms and improve cognitive and motor performance. This could have positive and innovative implications for the development of movement therapy for developmental disorders as well as for the promotion of an active lifestyle and self-determined participation in physical activity. Additionally, it may raise awareness of skateboarding, one of the fastest-growing sports (37).

We chose the trend sport skateboarding not only for its socio-cultural impact but also for its influence on self-education and self-efficacy processes of its users (38). Skateboarding has undergone a major evolution with its recent designation as an Olympic sport (39). Skateboarding requires and trains muscular fitness, coordination, balance, and dexterity (40). However, unlike conventional sports, skateboarding has few rules and regulations, but a great freestyle character that allows practitioners to perform and practice as they like, whenever they like until the individual goal is reached, making it highly participation-focused. Further, skateboarding may help children strengthen their self-concept and improve their ability to correctly assess risks and reflect on their behavior. This equally requires concentration abilities and sensorimotor skills, and at the same time aids the children's learning process through feedback on the motor level (38).

The objective of our study was to investigate the impact of a 16-week skateboarding intervention on motor and cognitive abilities as well as symptoms of ADHD in school-aged children. Intervention effects were analyzed by pre-post comparisons of three main areas, namely static and dynamic balance ability, concentration and attention capacity, and ADHD symptoms. We hypothesized that moreover, correlations between parameters of cognitive and motor ability could suggest that skateboarding benefits children on a holistic level if improvements in motoric performance led to improvements in cognitive capacity and vice versa. A further aim of this project was to enhance children's enjoyment of movement and physical activity by improving their ability to focus on a task for a longer period and increasing their frustration tolerance without relying solely on medication. This paper is therefore of high relevance as it may provide valuable insight into the chances and limitations of an informal sport like skateboarding to manage the symptoms of ADHD and develop autonomy and self-esteem. To quantify these aspects, the following hypotheses have been tested:

(1) Children with ADHD perform worse in cognitive and motor assessments compared to age-related neurotypical controls prior to the intervention.
(2) Children with ADHD show stronger improvements in cognitive, motor, and symptom assessments after participating in the skateboarding intervention compared to the ADHD control group that did not participate in the intervention.
(3) There is a significant correlation between the diverse parameters of motor ability, cognitive ability, and symptom expression at follow-up testing.

## 2 Methods

### 2.1 Participants

A total of 96 children from the area of Münster, Germany was initially recruited for the research study through announcements in schools, newspaper ads, social media advertisements, the district government of Münster and publications via the Skate Aid homepage, especially cooperation partner Titus Dittmann. 38 individuals were unable to attend their follow-up appointment due to personal reasons or the COVID-19 pandemic. 58 subjects in three different experimental groups participated voluntarily in all parts of the study and were eligible for data analysis.

The main intervention group (PR) consisted of 34 children (age: 11.3 ± 1.8 years, body height: 148.7 ± 12 cm, weight: 38.9 ± 10.1 kg) diagnosed with ADHD who participated in a skateboarding intervention. To control for the aging effect, one control group (PRCO) comprised children with ADHD who did not participate in the intervention (*N* = 14) (age: 10.8 ± 1.7 years, body height: 149.8 ± 11.5 cm, weight: 42.2 ± 11.7 kg). A second control group (ProKo) consisted of healthy children who participated in the skateboarding intervention (*N* = 10) (age: 9.9 ± 1.2 years, body height: 145.3 ± 9.8 cm, weight: 37.7 ± 9.3 kg). The group allocation of children with ADHD to the control or intervention group was randomized.

All children were between the ages of 8 and 13 years and had no professional experience in skateboarding. All ADHD children had been diagnosed by experienced pediatricians prior to the study. Medication with methylphenidate was documented and kept stable during the study. A majority of the subjects were male (*m* = 51/*f* = 7). The study was approved by the University of Münster ethics committee of faculty 7 (2018-10-HW), and written informed consent was obtained from the legal guardian of every participant.

### 2.2 Qualisys motion capture system

The kinematic data was collected using the Qualisys motion capture system and Qualisys Track Manager software (Oqus 500, Qualisys AB, Goeteborg, Sweden, 2018–2022 versions). In our study, the system involved 18 highspeed infrared cameras detecting the positions of passive optoelectronic reflective markers with a sampling rate of 200 Hz. The marker set consisted of a total of 64 reflective markers, each 20 mm in diameter, 59 of which were attached to anatomical landmarks using a modified version of the Helen Hayes marker set (Supplementary Material File 1). Five floor markers indicated start and finish positions for the motor tasks. For the kinetic data, eight stationary force plates (Kistler Group, Sindelfingen, Germany), 90 × 60 cm in size, recorded ground reaction forces and center-of-pressure positions at 1,000 Hz. The force plates and the kinematic system were synchronized with an external trigger signal (Qualisys AB, Goeteborg, Sweden).

### 2.3 Test procedure

**Figure 1:**
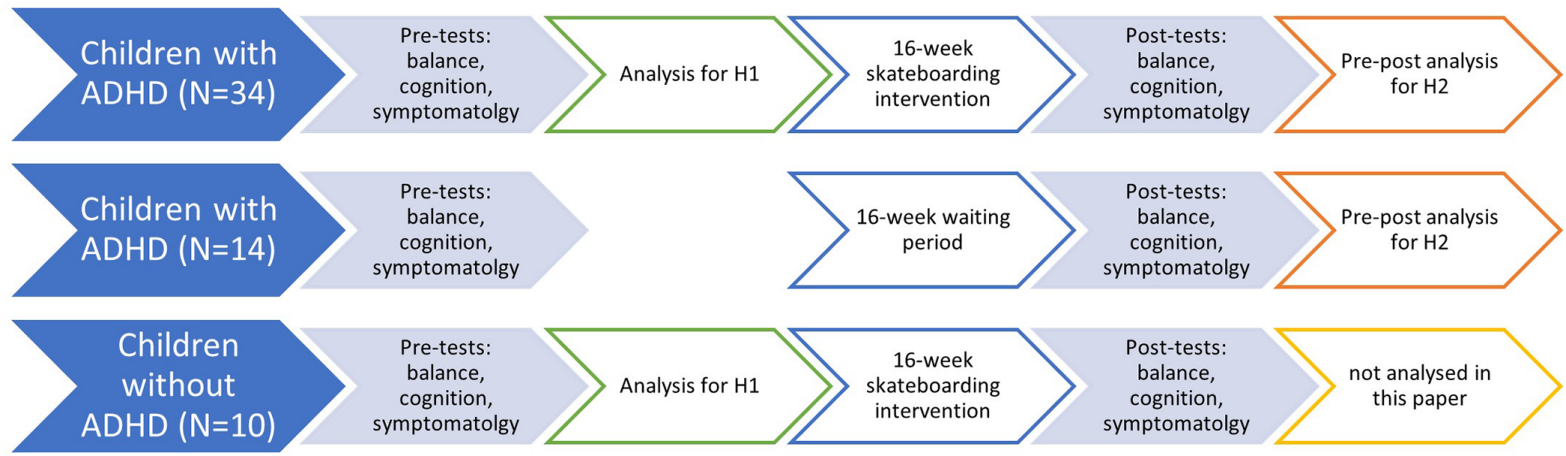
Project flow diagram.

This interventional study utilized a pre-pos*t*-test experimental design. The children and their parents visited the movement analysis lab of the University of Münster for pre- and post-measurements, which were conducted 16 weeks apart. The test protocol included a selection of movement tests to measure static and dynamic balance abilities. Furthermore, the subjects completed tests for cognitive performance, while parents filled out questionnaires to quantify symptomatology. Prior to the measurements, all parents attended a meeting to receive information and become familiar with the research study. For the pre-intervention examinations, subjects reported to the movement lab where they were provided with detailed study information. Parents, respective legal guardians were asked to provide written informed consent. Anthropometric data, including height and weight were collected while wearing sports clothing and no shoes An overview of the project flow is provided in Figure 1.

#### 2.3.1 Kinematic motor tests

To assess accuracy and precision in motor performance, we developed a test battery analyzing postural control specifically for this project, as there are no standardized protocols available. We regard the preciseness of our test battery as superior to other commonly used motor tests such as the MABC or BOTMP. After reflective markers were attached to the child, tasks on postural control were performed. For the single-leg stand, subjects were asked to stand as still as possible on the force plate on one leg for 25 s, alternating between legs. For the precision jumps, the aim was to execute a standing long jump, landing as close as possible to a target line. Start and finish lines were taped on the force plates and furnished with reflective markers. For the double-leg jumps, the lines were 90 cm apart. Requirements were to take off and land with both feet at the same time. For single-leg jumps, a 40 cm jump was required. Each child performed five trials for each condition (left, right, both) and was asked to remain standing at the landing position. For the subsequent balance task, subjects were asked to balance across a five-centimeter-wide, five-centimeter-high, and five-meter-long balance beam that was fixed to the force plates. Six trials were conducted for this task, with participants randomly assigned to start with either their left or right foot. When subjects lost balance and fell from the bar, they were instructed to resume the test from the position where they fell off.

#### 2.3.2 Cognitive tests

The subsequent assessment consisted of cognitive tests, specifically the Stroop color-word interference test and the d2-test. These tests are standardized test procedures for cognitive functioning, and evaluate selective attention and cognitive inhibition (Stroop test), sustained attention and working memory (d2-test), next to processing speed, visual scanning, and cognitive flexibility (41, 42). In the Stroop test, children were asked to quickly name the color of a colored word regardless of the word's meaning (the name of a color). In the d2-test subjects were asked to quickly cross out the letter “d” whenever it appears with two dashes above and/or below it. The overall number of edited letters per line and the number of errors made were evaluated.

#### 2.3.3 Questionnaires on symptom expression

The Child Behavior Checklist was used to detect developmental, behavioral, and emotional problems in all children. Children with intellectual disability were excluded from our study. To assess ADHD-symptomatology in all children the ADHD self-assessment and observer- assessment (parent) questionnaires of the DISPYS-II (Diagnostik-System für psychische Störungen) were used in both sessions.

### 2.4 Skateboarding intervention

During the four-month intervention period, children of the PR and ProKo groups participated in the skateboarding intervention, while the PRCO group represented a wait-list control group. The intervention comprised a weekly skateboarding workshop that was supervised and instructed by skate coaches from the local non-profit organization “Skate Aid”. The sessions lasted about 90 min and were held in an atmosphere that was neither therapeutic nor training oriented, but rather an opportunity for the children to autonomously shape the intervention by learning to skate and do the tricks that they wanted to do in a safe and supervised environment. The workshop was designed to assist children in problem-solving and goal attainment while skateboarding, thus helping to increase their self-confidence and reduce disruptive behavior. The skateboard and safety equipment were provided by Skate Aid throughout the study, giving the children an opportunity to continue skateboarding in their free time. Subjects were asked at the post-test appointment whether and how often they engaged in additional skateboarding, and how often they were unable to attend the weekly workshop. Subjects were free to pursue their usual leisure activities such as other sports or hobbies.

### 2.5 Data processing

#### 2.5.1 Motor performance data

The 3D-positions of the reflective markers were manually processed using the motion tracking software (Qualisys, Sweden), and every marker on the subject was labeled. Subsequently, the recorded sequences were cut, so that only the sequence with the required movement was analyzed. Edited data files were then exported to Matlab to extract kinematic information from marker positions and dynamic information from the force plates.

A custom periodic median filter was applied to the force plate data to remove humming noise. The marker and force plate data were filtered using a Butterworth filter with a cutoff frequency of 20 Hz and 40 Hz, respectively, to remove high-frequency noise. The force plate data were used to calculate the center of pressure (COP) as either equal to the COP of a single foot touching the ground or as the spatial average of the COPs of both feet touching the ground. The following motor performance measures were calculated from the data:

• Balance: Temporal average over the distance of the COP from the center line of the balance beam.
• One-leg stand: Temporal average over the distance of the COP from the barycenter of the COP trajectory.
• Precision jumps: The distance of the landing position from the target line. The landing position was defined as the position of the marker attached to the big toe of the landing foot at the time of landing.

All motor performance measures were referred to as “target error”, with lower values representing better performance.

#### 2.5.2 Cognitive performance data

Several parameters were calculated from the d2 cognition test, of which “completed targets” and “concentration capacity” were used in the statistical analysis. The number of completed targets gives information about the test processing speed, while the concentration capacity is calculated by subtracting the missed and falsely marked errors from the targets identified. In both cases, a higher value indicates a better performance. For the Stroop task, the word-color interference board was used and the time to completion was recorded, with lower values indicating better performance.

#### 2.5.3 Symptomatic data

From the symptom questionnaires, separate mean scores per child were calculated for attention deficit and hyperactivity, with lower scores indicating better results in both cases.

### 2.6 Statistical analysis

Statistical analyses were performed in MATLAB (version 2023a, The MathWorks, Inc., Natick, Massachusetts, USA). We used generalized linear mixed model (GLMM) analysis rather than traditional analysis of variance (ANOVA) for three main reasons. First, we had uneven group sizes due to dropouts. An ANOVA would have forced us to either discard data to balance the dataset or average across trials and subjects, both of which would have resulted in a significant loss of statistical power. Second, the complex mixture of within- and between-subject variables, as well as a variation of categorical variables and continuous covariates were more adequately accounted for with GLMM by incorporating random effects that capture individual variability within nested groups. In addition to random intercepts, we included random slopes, which further improved the goodness of fit. Third, GLMMs are much more robust to non-normally distributed data. For the motor performance data, we used gamma distribution functions together with the inverse function as the (canonical) link function. For the cognitive data and symptomatology, we used a normal distribution function. All model fits were performed using restricted maximum pseudo-likelihood.

The dependent variables of the GLMM fits were the corresponding target error for each of the motor tasks “Balance”, “One-leg stand” and “Precision jump”, the variables “d2 completed”, “d2 concentration”, and “Stroop test” for the cognitive tasks, and the variables “Attention deficit” and “Hyperactivity” for the assessment of ADHD symptoms. For hypothesis H1, which tested the effect of ADHD on the dependent variables, we restricted the dataset to the skateboarding group and the time to t1 and used “ADHD” as an independent variable and “Age” as a covariate. For hypothesis H2, which tested the effect of time on the dependent variables, we restricted the dataset to the ADHD group and used “Time” and “Skating” as independent variables, and “Age” and “Medication” (whether subjects indicated to use methylphenidate or not) as covariates. It should be noted that the term “skating” used in figures and tables always refers to the skateboarding intervention. While the independent variables were also tested for mutual interaction effects, this was not possible for the covariates, which thus entered the fit as purely additive terms.

After each GLMM fit, we performed multiple pairwise comparisons (post-hoc tests) based on the estimated marginal means using the “emmeans” package for Matlab (version 1.0.0 by John Hartman, available at https://github.com/jackatta/estimated-marginal-means). In essence, EMMs are the predicted means of the GLMM model for the data subpopulations under each combination of conditions. Because all *p*-values resulting from hypothesis tests of the GLMM fit, including post-hoc tests, are based on the estimated marginal means (EMMs), the data plots show these EMMs in the center of the violin plots, rather than the mean or median. To help interpret the results of the post-hoc comparisons, we calculated the percentage of change between pre- and pos*t*-testing for each pair, indicated by “%diff”.

For the analysis of the motor performance data, any point outside 1.5 times the interquartile range (0.25, 0.75), which sometimes resulted from artifacts, was considered an outlier, and removed before entering the GLMM analysis. That way, no more than 5% of the data were removed per fit (3.1% and 4.6% for hypotheses H1 and H2, respectively). Cognitive data and symptomatology were not affected by artifacts and therefore no outlier removal was performed. The quality of each model fit was checked using diagnostic plots, in particular checking for normal distribution of residuals and homoscedasticity.

For hypothesis H3, a partial correlation analysis was implemented using the statistics software JASP (version 0.18.3., Jasp Team, Amsterdam, Netherlands). To this aim, the data were first averaged per subject, so that for pre- and pos*t*-test data of *N* = 58 subjects, the degrees of freedom summed up to df = 114. Subsequently, a partial correlation analysis was performed on the averaged pos*t*-test data with the control variables being “Skating” (intervention group or control group), “Intervention” (before or after), “Age”, and “ADHD”. A partial correlation analysis removes correlations that are due to a common cause represented by a change in one or more control variables, rather than due to mutual dependencies. The variables “Skating”, “Intervention”, “Age”, and “ADHD” are plausible control variables for a change in the analyzed dependent variables, so they were included in the partial correlation analysis as conditional variables. Since the data were not normally distributed, we used Spearman's rho instead of Pearson's correlation coefficient r. The effect size of rho was interpreted in the same manner as Cohen's *r*, with the usual limits 0.1 = small, 0.3 = medium, 0.5 = large (43).

## 3 Results

### 3.1 Comparison between children with ADHD and controls

#### 3.1.1 Motor testing

**Figure 2:**
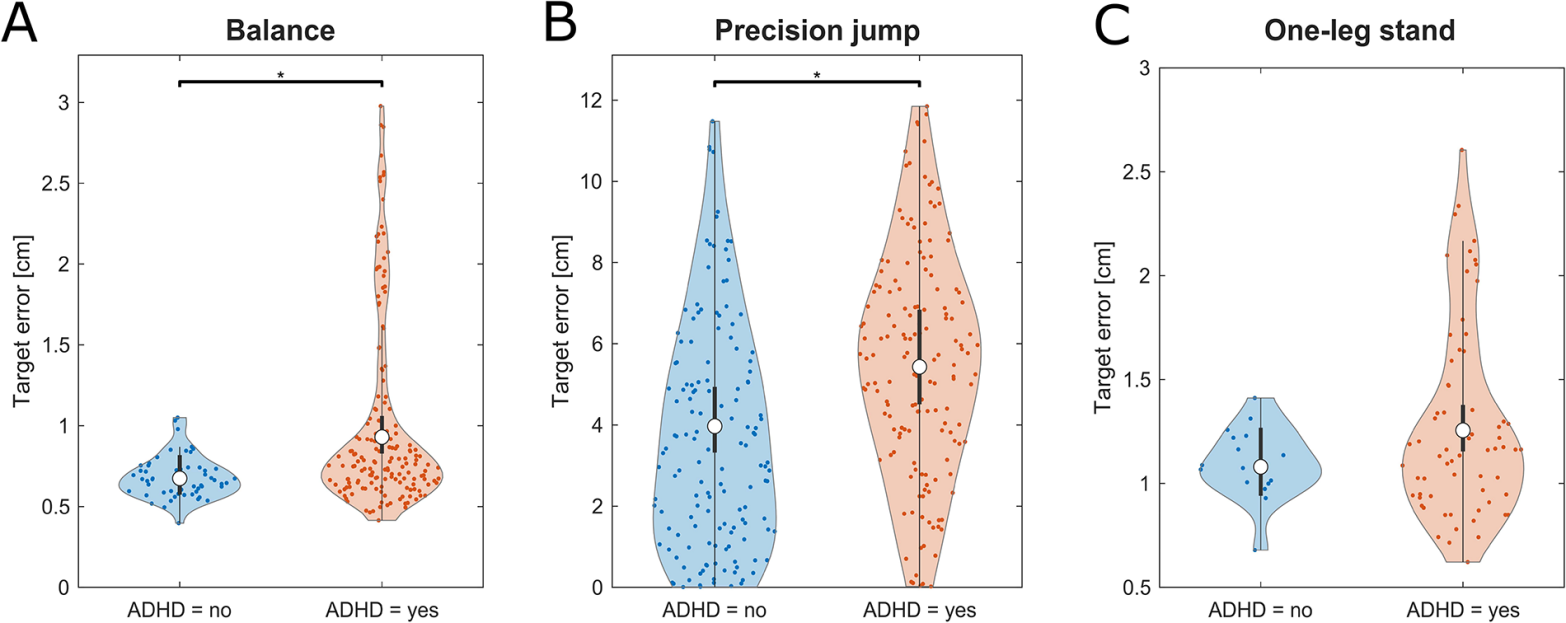
Violine plots of the linear model fit for motor tests on static and dynamic balance comparing children with and without ADHD. Lower values represent better performance. **(A)** Balance beam, **(B)** precision jump, **(C)** one-leg stand. Empty circles indicate the estimated marginal mean (EMM), error bars denote the 95% confidence interval. Brackets indicate significant differences, asterisks denote the corresponding level of significance: *p < .05, **p < .01, ***p < .001.

The linear model fit indicated that children with ADHD perform inferior in motor tests of static and dynamic balance than their unaffected peers. This finding was statistically significant for the balance (*F* = 7.02, *p* = .009, *η_p_*^2^ = 0.03) and the precision jump task (*F* = 6.1, *p* = .014, *η_p_*^2^ = 0.02). No significant differences between the groups were found in the single leg stand (Table 1; Figure 2). All descriptive statistics are provided in the Supplementary Material File 2.

**Table 1:** Results of the linear model fit comparing children with and without ADHD for all motoric and cognitive parameters.

	Term	*F*	*p*	*η_p_^2^*	Effect size
Balance beam	ADHD	7.02	**0**.009**	0.03	Small to medium
	Cov. Age	0.05	0.83	0.0002	Very small
Precision jumps	ADHD	6.1	**0**.014*	0.02	Small
	Cov. Age	0.01	0.98	0.00004	Very small
One-leg stand	ADHD	3.05	0.08	0.04	Small to medium
	Cov. Age	0.9	0.35	0.01	Small
d2-test completed targets	ADHD	9.35	**0**.004**	0.19	Large
	Cov. Age	41.42	**<0**.001***	0.5	Large
d2-test concentration capacity	ADHD	8.96	**0**.005**	0.18	Large
	Cov. Age	26.47	**<0**.001***	0.39	Large
Stroop test	ADHD	2.77	0.11	0.11	Medium to large
	Cov. Age	2.39	0.14	0.09	Medium to large

Significant results are in bold. Effect sizes are interpreted after Cohen's rule of thumb and signify how much of the effect can be explained by the given term.

#### 3.1.2 Cognitive testing

**Figure 3:**
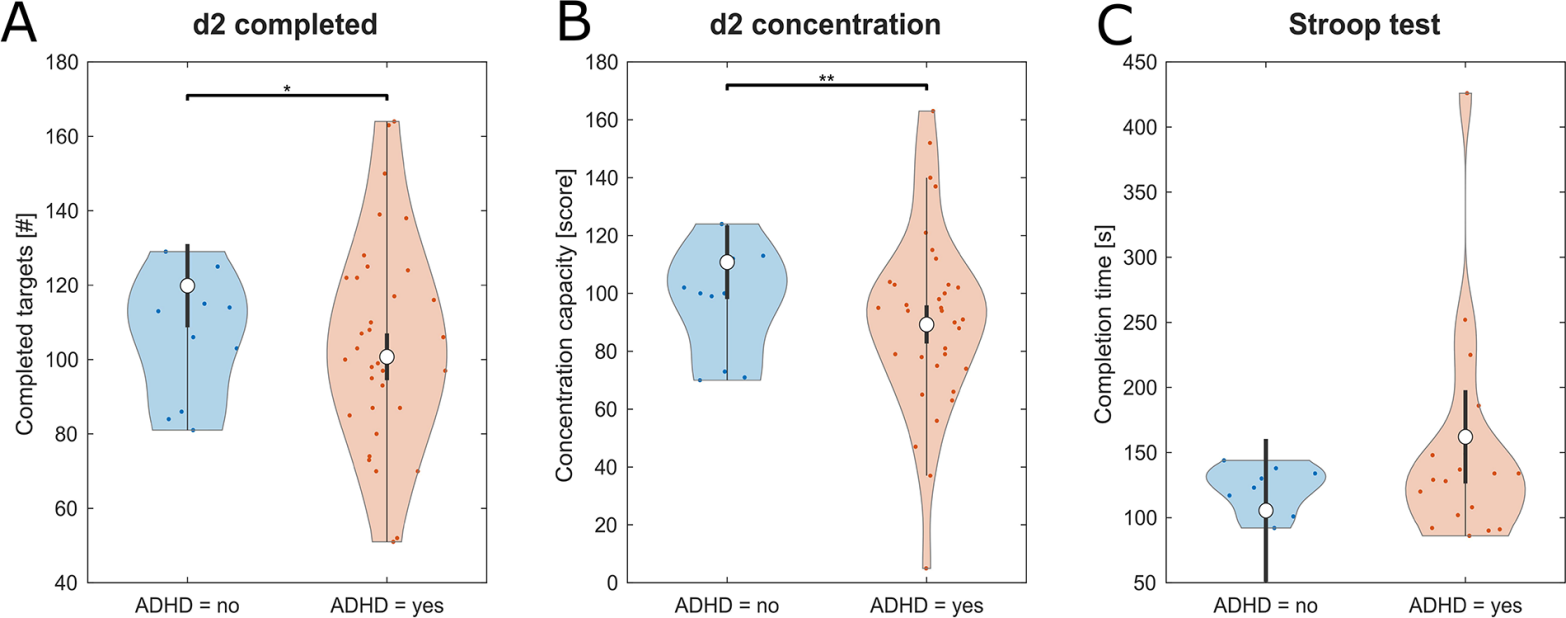
Violine plots of the linear model fit for cognitive tests on attention-focusing skills comparing children with and without ADHD. Higher values represent better performance for **(A)** d2-test number of completed targets and **(B)** d2-test concentration capacity and lower values representing better performance for **(C)** Stroop test. Empty circles indicate the estimated marginal mean (EMM), error bars denote the 95% confidence interval. Brackets indicate significant differences, asterisks denote the corresponding level of significance: *p < .05, **p < .01, ***p < .001.

Children with ADHD performed inferior in the d2-test compared to their unaffected peers. This finding was statistically significant for both d2-test parameters, number of completed targets (*F* = 9.35, *p* = .004, *η_p_*^2^ = 0.19) and concentration capacity (*F* = 8.96, *p* = .005, *η_p_*^2^ = 0.18). Moreover, “Age” had a strongly significant effect on the outcome. No significant differences between groups were found for the Stroop test (Table 1; Figure 3).

### 3.2 Effect of skateboarding on children with ADHD

#### 3.2.1 Motor testing

**Figure 4:**
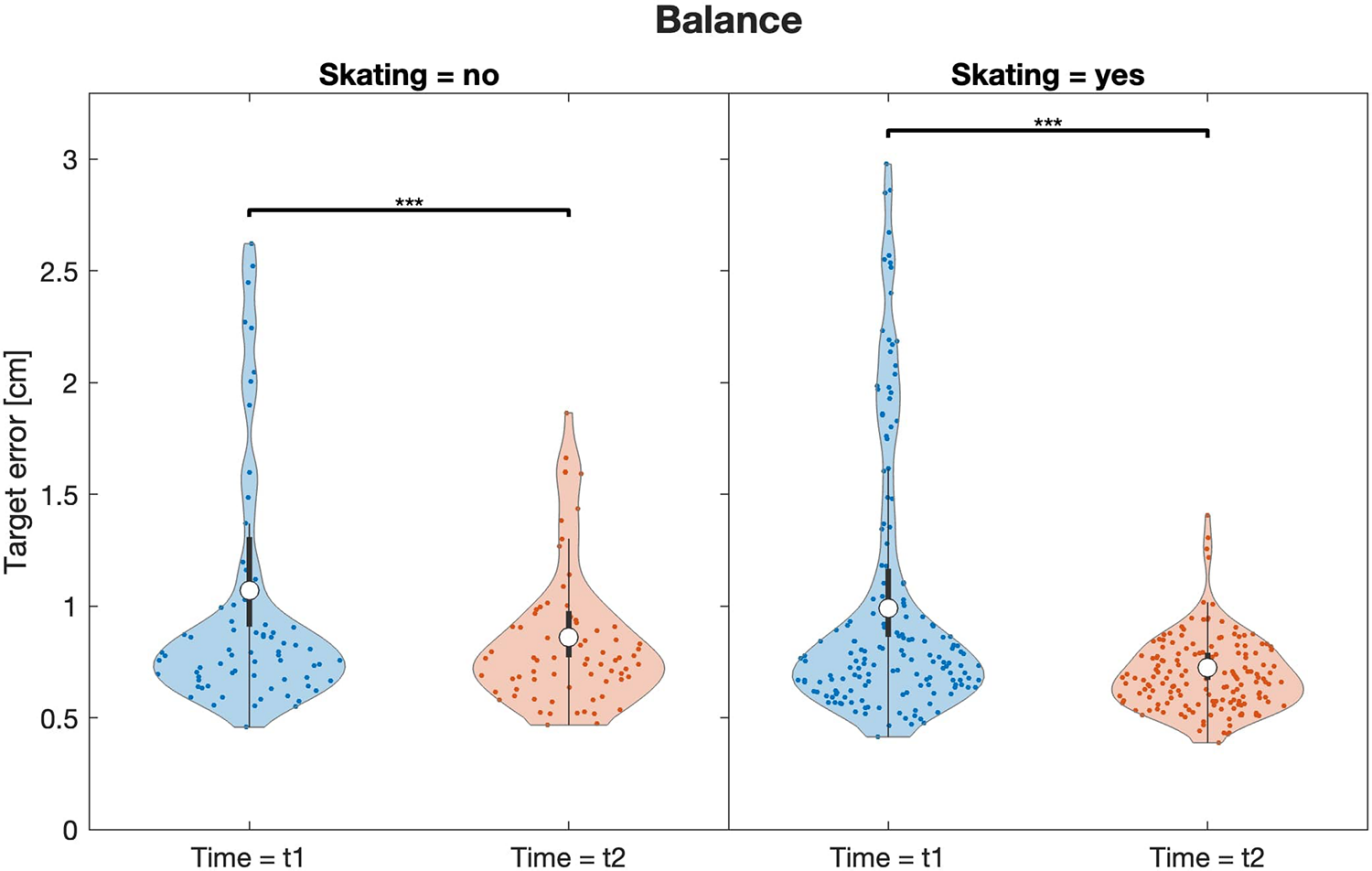
Violine plots of the linear model fit for the motor test on dynamic balance on the balance beam comparing intervention and control group. Lower values represent better performance. Empty circles indicate the estimated marginal mean (EMM), error bars denote the 95% confidence interval. Brackets indicate significant differences, asterisks denote the corresponding level of significance: *p < .05, **p < .01, ***p < .001.

ADHD-affected children performed better in the balance beam task after participating in the four-month skateboarding workshop compared to the age-matched ADHD-affected control group without skateboarding intervention (*F* = 8.17, *p* = .004, *η_p_*^2^ = 0.02) (Table 2). A consequent post-hoc analysis showed that the skateboarding group improved their balance performance significantly (*F* = 38.9, *p* < .001, *η_p_*^2^ = 0.08) (Table 3; Figure 4) with medium effect size, while the waitlist-control group underwent small to medium changes. The results for one-leg stand and precision jumps yielded no significant results (see Supplementary Material File 3). Descriptive statistics of all three assessment areas are provided in the Supplementary Material File 4.

**Table 2:** Results of the linear model fit comparing intervention and control group for motoric, cognitive, and symptomatic items.

	Term	*F*	*p*	*η_p_^2^*	Effect size
Balance beam	Time	20.2	**<0**.001***	0.04	Small to medium
	Skating	0.87	0.35	0.002	Very small
	Cov. Age	0.01	0.92	0.00002	Very small
	Cov. Medication	5.64	**0**.02*	0.01	Small
	Time × Skating	8.17	**0**.004**	0.02	Small
d2-test completed targets	Time	26.26	**<0**.001***	0.23	Large
	Skating	2.07	0.15	0.02	Small
	Cov. Age	30.97	**<0**.001***	0.26	Large
	Cov. Medication	1.25	0.27	0.01	Small
	Time × Skating	7.21	**0**.009**	0.07	Medium
d2-test concentration capacity	Time	41.2	**<0**.001***	0.31	Large
	Skating	2.52	0.12	0.03	Small to medium
	Cov. Age	21.82	**<0**.001***	0.2	Large
	Cov. Medication	0.58	0.45	0.006	Small
	Time × Skating	3.36	0.07	0.04	Small to medium
Stroop test	Time	9.37	0.004 **	0.16	Large
	Skating	3.77	0.06	0.07	Medium
	Cov. Age	14.74	**<0**.001***	0.23	Large
	Cov. Medication	0.19	0.66	0.004	Very small
	Time × Skating	2.4	0.13	0.05	Small to medium
Attention deficit	Time	25.51	**<0**.001***	0.22	Large
	Skating	1.3	0.26	0.01	Small
	Cov. Age	0.17	0.69	0.002	Very small
	Cov. Medication	0.001	0.97	0.00001	Very small
	Time × Skating	1.2	0.28	0.01	Small
Hyperactivity	Time	9.4	**0**.003**	0.1	Medium to large
	Skating	1.26	0.26	0.01	Small
	Cov. Age	2.54	0.11	0.03	Small to medium
	Cov. Medication	3.42	0.07	0.04	Small to medium
	Time × Skating	0.02	0.9	0.0002	Very small

Significant results are in bold.

Only significant parameters are shown here. Effect sizes are interpreted after Cohen's rule of thumb. Asterisks denote the corresponding level of significance: *p < .05, **p < .01, ***p < .001.

**Table 3:** Results of the post-hoc analysis comparing times 1 and 2 of the two different experimental groups for motoric, cognitive, and symptomatic items displayed in Table 2.

	Skating	*p*_corr_	%diff	*F*	*η_p_* ^2^	Effect size
Balance beam	No	**<0**.001***	−19.57	20.2	0.04	Small to medium
	Yes	**<0**.001***	−26.85	38.9	0.08	Medium
d2-test completed targets	No	**<0**.001***	10.35	26.3	0.23	Large
	Yes	**<0**.001***	17.65	51.9	0.37	Large
d2-test concentration capacity	No	**<0**.001***	13.79	41.2	0.31	Large
	Yes	**<0**.001***	20.07	57.9	0.39	Large
Stroop test	No	**0**.004**	−21.03	9.37	0.16	Large
	Yes	**0**.001**	−21.66	13.88	0.22	Large
Attention deficit	No	**<0**.001***	−15.21	25.51	0.22	Large
	Yes	**<0**.001***	−19.54	32.83	0.27	Large
Hyperactivity	No	**0**.006**	−15.91	9.4	0.1	Medium to large
	Yes	**0**.004**	−18.96	8.93	0.09	Medium to large

Significant results are in bold.

Statistically corrected *p*-values are indicated by pcorr. Relative changes are indicated in percent by “%diff”. Effect sizes are interpreted after Cohen's rule of thumb. Asterisks denote the corresponding level of significance: *: *p* < .05, **: *p* < .01, ***: *p* < .001.

#### 3.2.2 Cognitive testing

**Figure 5:**
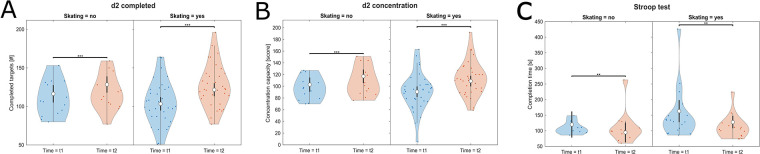
Violine plots of the linear model fit for cognitive tests on attention-focusing skills comparing intervention and control group. Higher values represent better performance for **(A)** d2-test completed targets, **(B)** d2-test concentration capacity, and **(C)** Stroop test. Empty circles indicate the estimated marginal mean (EMM), error bars denote the 95% confidence interval. Brackets indicate significant differences, asterisks denote the corresponding level of significance: *p < .05, **p < .01, ***p < .001.

ADHD-affected children performed better in the d2-test after participating in a four-month skateboarding workshop compared to the age-matched ADHD-affected control group without skateboarding. The significant interaction effect for completed targets (*F* = 7.21, *p* = .009, *η_p_*^2^ = 0.07) and the respective main effect Time (*F* = 26.26, *p* < .001, *η_p_*^2^ = 0.23) showed that the two groups were affected differently (Table 2). A consequent post-hoc revealed that both groups underwent significant improvement, however with a larger percentage of improvement for the skateboarding group (Table 3). For concentration capacity in the d2-test, a significant main effect was found (*F* = 41.2, *p* < .001, *η_p_*^2^ = 0.31) (Table 2). Again, the respective post-hoc analysis showed that both groups improved significantly, with a greater improvement for the skateboarding group (Table 3). For time needed for Stroop test completion, a significant main effect was found (*F* = 9.37, *p* = .004, *η_p_*^2^ = 0.16) (Table 2). The corresponding post-hoc analysis showed that both groups improved significantly and to an almost identical degree (Table 3). For a graphical presentation refer to Figure 5. Furthermore, “Age” had a strongly significant effect on all three cognitive test outcomes.

#### 3.2.3 Symptom expression

**Figure 6:**
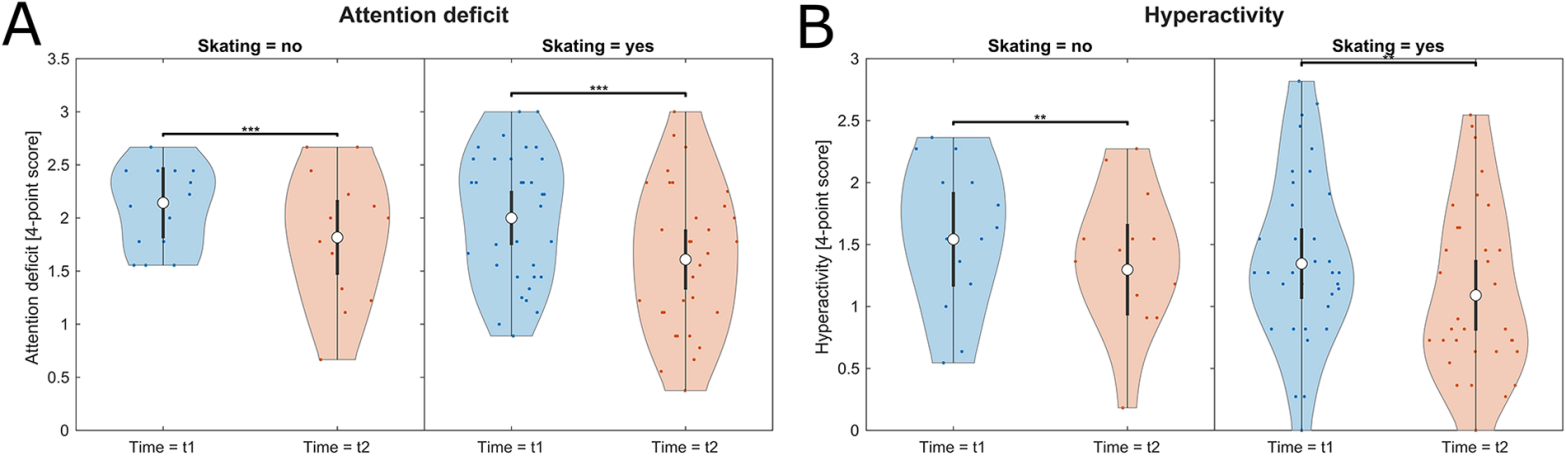
Violine plots of the linear model fit for symptom expression comparing intervention and control group. Lower values represent better scores. **(A)** Attention deficit, **(B)** Hyperactivity. Empty circles indicate the estimated marginal mean (EMM), error bars denote the 95% confidence interval. Brackets indicate significant differences, asterisks denote the corresponding level of significance: *p < .05, **p < .01, ***p < .001.

ADHD-affected children presented significantly lower signs of attention deficit and hyperactivity after participating in a four-month skateboarding workshop compared to the age-matched ADHD-affected control group without skateboarding. Significant results for the main effect Time for attention deficit (*F* = 25.51, *p* < .001, *η_p_*^2^ = 0.22) and hyperactivity (*F* = 9.4, *p* = .003, *η_p_*^2^ = 0.1) (Table 2) showed that a development on the symptom level occurred between pre- and pos*t*-tests. A consequent post-hoc analysis revealed that both experimental groups improved their symptom scores very significantly with large effect sizes. For both symptoms, the intervention group, however, achieved a stronger improvement percentage wise (Table 3; Figure 6). The covariates “Age” and “Medication” did not influence symptom expression.

### 3.3 Correlation between cognitive functioning, motor abilities, and symptomatology

The correlation coefficient Spearman's rho has been computed to assess the relationship between the diverse parameters of motor ability (balance, precision jumps, one-leg stand), cognitive ability (d2-test number of completed targets, d2-test concentration capacity, Stroop test), and symptom expression (attention deficit, hyperactivity) on the pos*t*-test data of all groups, with a partial out of “Age”, “ADHD”, “Intervention” and “Skating”. Here we focus on the partial correlation, where potential common cause effects of the variables “Age”, “ADHD”, “Skating”, and “Intervention” have been removed. The partial correlation gives a better estimation of mutual dependencies between variables than the standard correlation (see Methods section for details). For the sake of completeness, a standard correlation analysis is provided in the Supplementary Material Files 5, 6.

As expected, there was a moderate positive correlation between the two symptom variables hyperactivity and attention deficit, as well as a strong positive correlation between the two variables of the d2-test, next to a moderate negative correlation between both d2-test variables and the Stroop test. A weak positive correlation was found between static and dynamic balance ability for the one-leg stand and the balance beam task.

**Figure 7:**
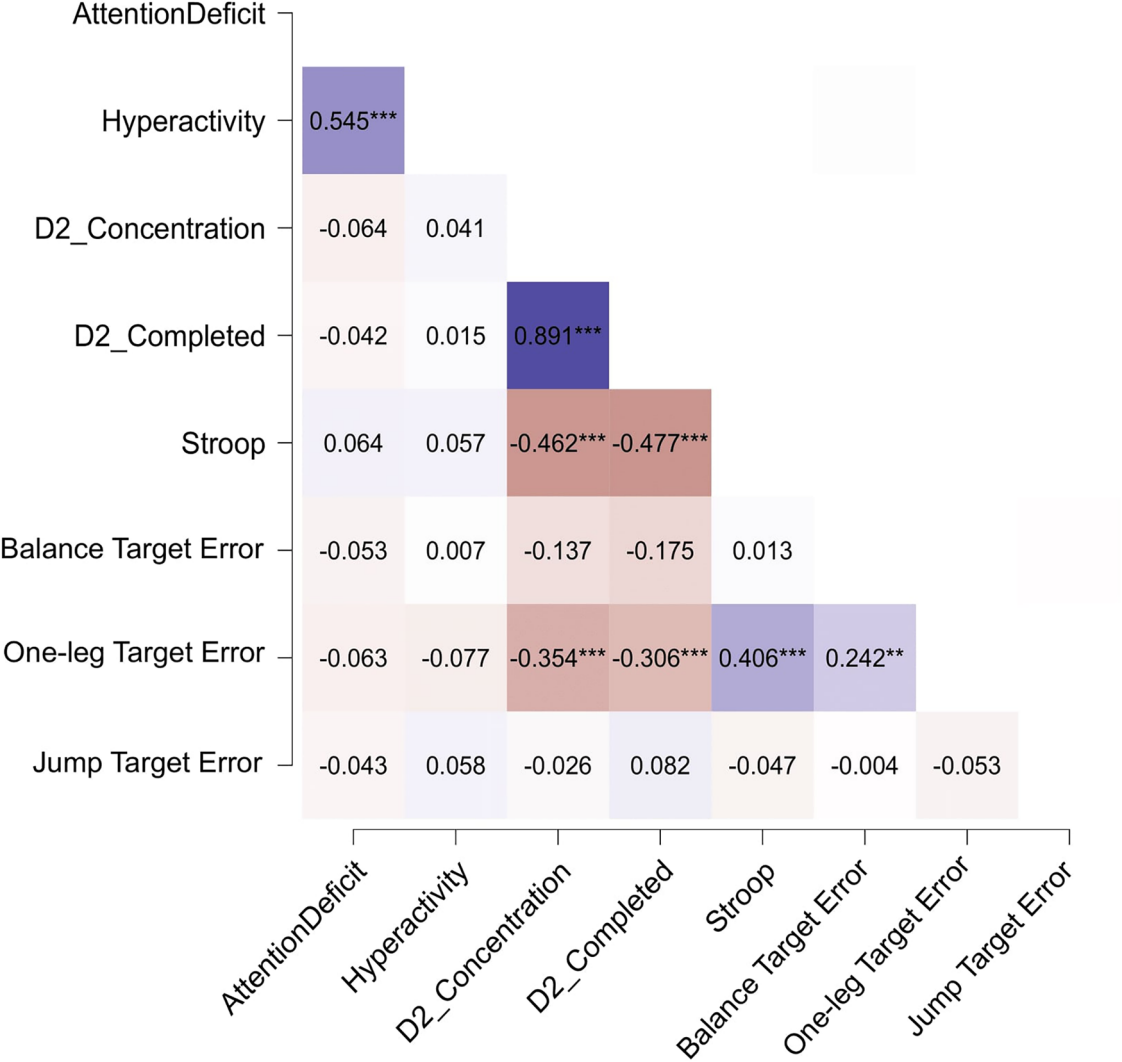
Heatmap graphically representing the results of the partial correlation analysis. Darker fields correspond to stronger correlation, bluish and reddish fields correspond to positive and negative correlation, respectively. Correlation is measured in terms of Spearman's rho. Asterisks denote the corresponding level of significance: *p < .05, **p < .01, ***p < .001.

For *N* = 58 subjects, there were weak to moderate correlations between static balance ability in the one-leg stand and the tests of cognitive ability, for d2 concentration capacity, rho = −0.354, *p* < .001, d2 number of completed targets, rho = −0.306, *p* < .001, and Stroop test, rho = 0.406, *p* < .001 (Figure 7, results in table form are provided in the Supplementary Material File 7).

## 4 Discussion

### 4.1 Discussion of results

The aim of this paper was to investigate whether a four-month skateboarding workshop can improve symptoms of attention deficit and hyperactivity in ADHD-children, as well as postural control in terms of static and dynamic balance, and performance on the d2-test and the Stroop test.

We first confirmed a difference in cognitive and motor abilities between children with and without ADHD with the patient group presenting worse results than the controls. Children without ADHD performed significantly better in the balance beam and precision jump tasks as well as in both d2-test parameters compared to ADHD-affected children. Detriments in balance and postural control have commonly been observed in ADHD patients (44–48). In a balancing platform task, the ADHD group did not only present significantly greater sway amplitudes in all trials compared to the group of typically developing children, they also showed stronger deterioration of balance performance with an increasing number of trials which may be explained by delayed cerebellar development (48). Moreover, Ghanizadeh (47) reported the disturbed inhibition of excessive movement and sensory processing problems that arise as a co-occurring condition with ADHD and negatively contribute to balance and coordination ability. An experiment by Shum and Pang (46) substantiated the ADHD group's deficits in standing balance with disruption of sensory signals. They identified especially the visual system to be involved in the contribution of balance deficits (46).

Likewise, inferior performance of the ADHD group in cognitive tasks has been reported in the literature before. It can be attributed to the core symptom of attention deficit, especially over a longer period of time (49), but also to impairments in executive functions (5). Shaw and colleagues (50) suggest that a delay in cortical maturation in the prefrontal regions is responsible for such deficits, as these areas are crucial for the control of cognitive processes such as attention and motor planning. Those significant differences were found despite both, the group of children with ADHD and the control group were fairly heterogenous with children of all school forms and all ages between eight and 13 present. While the children's education level was surveyed, no information on the parents' education level, or socioeconomic status was available.

The static balance assessment and the word-color interference (Stroop) test did not appear to discriminate between the presence or absence of an ADHD diagnosis.

The second and central hypothesis yielded mixed results regarding the usefulness of a skateboarding intervention for children with ADHD. In terms of motor outcomes, only the balancing task showed a significant interaction effect with greater improvement for the skateboarding group. While both the skateboarding and non-skateboarding ADHD groups significantly improved their balance performance, a stronger effect size and a larger percentage of improvement were observed for the skateboarding group. No differences were found between the skateboarding and waitlist-control groups on the one-leg stand and the precision jump tasks. From a movement science perspective, improvements in all three motor tasks were expected due to the practical and movement-oriented intervention program. For the here investigated tasks of static balance and precision jumping, the skateboarding intervention did not result in an improvement. Conversely, for dynamic balance skills, skateboarding did help children to achieve greater progress than that resulting from development and maturation alone, as highlighted by the 20% vs. 27% gains in the control and intervention groups, respectively. It is plausible that the similarity in motor demands between skateboarding, which is characterized by maintaining and restoring balance during positional changes (51), and a balance task such as walking over a narrow beam contributed to this development (40). In addition, certain relevant brain regions are stimulated during the intervention sessions. The phenomenon of whether a sport like skateboarding can remodel neuronal structures of the brain has been investigated in a bachelor thesis (52). Skateboarding-experienced subjects were found to show increased activity in motor-relevant areas when watching skateboard tricks due to active mirror neurons (52). Another interesting finding is the small, but significant effect of medication use on balance performance in the present study, suggesting that methylphenidate use is particularly helpful for motor coordination tasks (53, 54) as well as postural stability and balance (55, 56), as supported by the literature. Improving balance and motor coordination in general provides a significant benefit to children with ADHD, as it could positively impact their well-being and daily life by minimizing the risk of injury (46, 57).

Although a movement intervention was employed, there was more progress in cognition and symptoms than in motor skills. Both attention tests exhibited main effects of time, indicating that cognitive performance improved significantly for the skateboarding and non-skateboarding ADHD groups over time. A learning effect in both groups for this type of test may be possible. However, the post-hoc test showed that a larger percentage of improvement was observed for the skateboarding group (18% improvement) compared to the control group (10% improvement) in terms of completed d2 targets. This suggests that a sport like skateboarding may further enhance the favorable effects of medication, maturation, and aging on concentration and test completion time. Comparably, a study by Magistro and colleagues (58) found that a long-term physical activity intervention with a conventional sport like football can enhance cognitive performance and executive functions, particularly in attention-focusing skills, processing speed, and cognitive flexibility. The findings are consistent with functional magnetic resonance imaging (MRI) research, which demonstrated that regular physical activity during childhood can enhance prefrontal cortex function involved in cognitive control (59).

In both the d2-and Stroop tests, the covariate “Age” was found to be highly significant. This indicates that age has a profound influence on the outcome of these tests, which is a common finding in cognitive testing (60, 61). However, it is important to note that the cognitive test results of this study were not standardized to age-matched norm values, which likely influenced the results. Standardization of the results for motor and symptom areas was not possible, thus this step was omitted. Surprisingly, the covariate “Medication” did not significantly affect the test outcome. This contrasts with the findings of Brodeur and colleagues (60), who reported that children taking methylphenidate perform better in a selective attention task.

In terms of symptom expression, significant main effects for Time were observed. Both attention deficit and hyperactivity were significantly reduced after the four-month period for both the skateboarding and the control group. The post-hoc analysis revealed that the skateboarding group reduced symptom manifestation more notably than their peers who did not take part in the intervention. However, with a 15% vs. 20% reduction in attention deficit for the control and intervention groups, respectively, the difference between the two groups, i.e., the intervention effect, is not as substantial as expected. Even though, according to effect sizes, the improvements were large, the difference in reduction of hyperactivity between the control group (16%) and the intervention group (19%) is rather small. It seems that a four-month period of aging and development alone has already brought an improvement in symptom expression. The additional effect of 3%, attributable to the skateboarding intervention is small, yet measurable. Since ADHD is a spectrum disorder, we can assume that the intervention effect but also the age and development effect took on various dimensions among the subjects. Besides this, clinical characteristics are likely to have an influence on the extent a child is affected by an intervention. A few parents were open towards sharing clinical characteristics of their child, which was however not compulsory. We therefore know that in few individual cases anxiety or child depression were present. In this context, it is striking that the covariate “Medication” did not seem to have a significant influence on the present assessment. Age difference, as another covariate, also had no significant impact on symptom expression. This is consistent with the fact that although ADHD symptoms may be more or less intense at different phases throughout childhood and young adolescence (62), they commonly persist until adulthood.

In contrast to motor and cognitive measurements, the symptom assessment was made by the parents rather than obtained from the child's own performance. The reported positive developments in both groups indicate, therefore, that improvements were pronounced enough to be recognized by the family as the closest environment. It has been shown that the parent-child relationship is an essential predictor for behavioral problems in children with ADHD and that a strong relationship has a great beneficial impact on the behavior of the child (63). Acquiring a new outstanding skill may additionally increase confidence and self-concept in children (64). Moreover, many parents reported how delighted their children were about the complete absence of heteronomy, which is another beneficial factor for children with ADHD (65). However, it must also be kept in mind that with a questionnaire as the data collection method, parents were potentially susceptible to answer socially desirable at follow-up, independent of whether their child took part in skateboarding or not.

In line with our findings, a recent meta-analysis shows that physical exercise has a significant positive impact on attention and executive function (66). Likewise, Jensen and Kenny (67) reported that hyperactivity and impulsive and aggressive behavior were reduced after a long-term yoga intervention. Ko and colleagues (68) suggest that swimming has the potential to alleviate ADHD symptoms by upregulating dopamine levels and downregulating dopamine D2 receptor expression. Overall, physical activity interventions may be a promising method for children with ADHD to manage symptoms due to their capacity to positively impact functional neurocognitive development (69).

Skateboarding as an ADHD intervention seems to have benefits similar to those of other established approaches that focus not on a specific, but various forms of physical activity and fitness (20, 21). Other unconventional ADHD therapy methods such as dance movement therapy or martial arts mindful movement therapy emphasize creativity, joy of movement and self-expression of children, while at the same time helping them cope with their symptoms (22–28), comparable to the skateboarding workshop of the present study. However, as opposed to the therapy forms mentioned above, our workshop does not have the claim to be perceived as a therapy, but rather as a voluntary intervention. Such approaches commonly have a more positive effect on intrinsic motivation and implicit learning of the participants (38).

In general, the literature reports that exercise programs are successful in improving both motor skills and executive functions in children with ADHD (70, 71). Therefore, it was hypothesized that the motor, cognitive, and symptom parameters examined here would be correlated, such that a positive development in one area would favor a positive development in the other. This hypothesis, however, is not supported by the data, as there was no significant correlation between items of different domains. Moderate to strong correlations between the symptom parameters or between cognitive test parameters were clearly expected and will not further be discussed.

Of particular interest were correlations between the motor and cognitive domains, and between the motor and symptomatic domains. However, only the one-leg stand was found to be moderately correlated with the attention-focusing tasks d2-test and Stroop test (Figure 7), when conditioned on the covariates “Age”, “ADHD”, “Skating”, and “Intervention”. Also, there was no significant correlation between symptom expression and motor ability. On the other hand, an unconditioned correlation analysis revealed that (at the disregard of the four covariates) weak to moderate correlations between motor and symptom areas arise (see Supplementary Material Files 5, 6). However, since we can assume that the covariates do have a decisive effect on the mutual correlation of the variables, we deem the interpretation of the partial correlation to be more useful.

Previous research on the relationship between core ADHD symptoms and motor performance found that impulse control and attention are essential predictors of fine and gross motor skills in children with ADHD (6). Similarly, there is evidence for the relationship between cognitive ability and symptom expression (12), as well as a connection between motor and cognitive function in ADHD patients (48). Based on the common evidence of cerebellar dysfunction, Goetz and colleagues (48) hypothesized that the dynamic balance impairment of children with ADHD would correlate with attention. They found that balance correlates with neuropsychological measures of reaction time consistency, likely due to the cerebellum's function as an integrative structure for balance control and cognition such as timing and anticipatory regulation (48). However, these findings could not be confirmed in our study.

During the four-month skateboarding workshop the ADHD-affected children demonstrated their ability to focus on learning a new sport skill through intrinsic motivation and interest as reported by the skate coaches. The challenging nature of the sport is likely to play an important role in the development of mental attributes such as discipline and perseverance (38). It is therefore conceivable that the children may be able to reproduce a similarly high level of attention and improved behavioral control in school and other everyday situations and apply it to tests of cognitive function, thus creating a reciprocal beneficial influence between the two domains when mediated by skateboarding. However, apart from the weak to moderate correlation between static balance ability and both cognitive tests, these considerations were not supported by the data. A possible reason may simply lie in the weakness of the employed test methods. In order to determine causal relationships, precise tests that are capable of capturing an ability in an isolated manner would be needed. This was not strictly warranted by the movement test battery, which through its design measured not only balance ability but also concentration and motivation, a limitation that is further discussed in the following section. We suppose that with a different choice of motor tests, the correlations with cognitive performance may be visible as it is known from the literature (6, 12, 48).

### 4.2 Limitations

ADHD is a spectrum disorder; we observed a wide range of symptoms. Additionally, our patient group was heterogeneous regarding the age span of eight to 13 years, as well as the high number of male subjects with a ratio of roughly 7:1. The latter is however in line with the typical gender ratio in clinical studies which is between 5:1 and 9:1 (72). Furthermore, the experimental groups varied considerably in size. The ADHD group that received the skateboarding intervention was by far the largest, with 34 subjects, whereas the two control groups, one with ADHD children who did not participate in the skateboarding intervention and another with neurotypical children who took part in the intervention included only ten and 14 children, respectively. To avoid losing statistical power by excluding data from the largest group, a GLMM analysis capable of handling unbalanced data was used instead of a traditional ANOVA, as explained in the methods section. Nevertheless, the considerable imbalance in group sizes poses a systematic threat to statistical power. For a future extension of this research, a control group should ideally consist of age- and gender-matched subjects.

Another significant limitation is the relatively small sample size. Correspondingly the overall sample size of 58 children does not allow any broad generalizations or represent the entire ADHD population, which was however not intended with this study. The present findings can be used to draw conclusions about the effect on girls and boys diagnosed with ADHD between the ages of eight and 13. A future effort would need to replicate this study with a larger and more diverse sample to allow general conclusions about the intervention's effect on children with ADHD. A more detailed collection of information on demographic and clinical characteristics of the patient group would be required as this may greatly enhance the applicability of the findings.

Another potential limitation is the wide range of neurobiological status among the participants. This study included children with and without regular medication intake. While it was assessed which children were under the influence of pharmacological measures at the time of the experiment, which could thus be modeled as a covariate, we do not have information on the exact dosage and type of medication used. Due to the heterogeneity of the disorder and the resulting differences in medication requirements, it was not possible to make a clear distinction between the medicated and unmedicated groups. Future research could use electroencephalography (EEG) or MRI to further explore differences in pathological profiles and the origin of possible cognitive adaptations. In terms of symptom assessment, an online form over a seven-day timespan may be more reliable to estimate symptom expression rather than a one-time questionnaire which has the limitation of being a momentary snapshot.

Further, attendance at the skateboarding workshops differed. While most subjects missed a maximum of two offered sessions, several children reported missing the workshop between four and six times. In addition, it was recorded how often the children skated in their free time, which ranged from more than once a week to once a month, making it very difficult to disclose how much of the effect can be attributed to skateboarding. Calculating a covariate from the amount of workshop participation together with the amount of additional skateboarding provides only moderately helpful knowledge, as the available information is very imprecise.

The present study design only controls for acute intervention effects but lacks long-term follow-up measurements. This is a substantial shortcoming as the sustainability of the intervention effect remains unclear. Future endeavors should include long-term follow-up measurements to quantify whether the therapeutic effects can be sustained with an intervention phase of four months or if the approach is only effective if practiced continually.

A final limitation concerns the test items used to measure balance ability. As mentioned above, this original test battery was chosen in the absence of a suitable standard balance test. A total of six trials were administered for the balance task and 15 trials for the precision jumps. During these measurements, it was easy to observe how the children became bored with these rather simple tasks and the high number of repetitions required. Since hyperactivity, inattention, and difficulty devoting to a task for long periods of time are among the core symptoms of ADHD, it was observed more than once that after the first few, often excellent, trials, the children became distracted and stopped trying. Instead of averaging over tasks, it was briefly considered to use only each subject's best performance for each task. However, this idea was abandoned as it interfered with the nature of the disorder and its symptoms. Future research in this area should therefore assess which performance measures are appropriate to reliably quantify motor skills, possibly by making the tests more task-specific and skateboard-related.

Finally, it is important to highlight some strengths of the present study such as the long-term intervention, the extensive statistical analysis and the interdisciplinary research team. Another noteworthy strength is certainly the joy of discovering and improving in the new sport that the participants experienced.

## 5 Conclusion

This study is the first to investigate the effects of a long-term skateboarding intervention on motor and cognitive performance as well as symptom severity in school-aged children with ADHD. Children with ADHD performed inferior in specific tests of motor and cognitive abilities compared to their healthy peers. A four-month skateboarding intervention has helped the patient group to significantly improve their dynamic balance ability, providing important consequences for injury risk and quality of life. Moreover, significant improvements in cognitive functioning and attention-focusing were found after the intervention. This has frequently been reported in previous research on movement therapies for ADHD patients and is likely related to enhanced prefrontal cortex functioning from regular exercise. Further, engaging in physical activity like skateboarding positively influenced ADHD core symptoms, which promotes a stronger parent-child relationship and, in turn may favor the child's self-concept and social behavior, as well as their discipline and perseverance in learning a new skill. Surprisingly, a symptom improvement of a similar extent was found even without skateboarding, meaning the effect attributable to skateboarding is considerably low, yet measurable.

The data did not support a reciprocal influence between motor and cognitive skills, nor between motor skills and ADHD symptomatology despite previous research reporting otherwise. Overall, the data are consistent with the idea that sports practiced out of intrinsic motivation has a positive impact on the development of children with ADHD, even if it cannot completely replace medication. The present study, particularly the feedback from the skate coaches, showed how quickly and enthusiastically the children embraced an informal sport like skateboarding, and that a four-month workshop encouraged them to self autonomously engage in physical activity. To retain the benefits, it is advisable to practice a sport on a long-term basis. All in all, skateboarding as a form of movement intervention can be considered an effective method for children with ADHD to deal with their symptoms and deficits. Thorough conceptualizations to implement this type of therapy await further research.

## Data Availability

The datasets presented in this study can be found in online repositories. The names of the repository/repositories and accession number(s) can be found below: https://doi.org/10.17879/26968596154.
