# Supplementary material for: The effects of a four-month skateboarding intervention on motor, cognitive, and symptom levels in children with ADHD
Source: Front Pediatr. 2024 Dec 2;12:1452851. doi: 10.3389/fped.2024.1452851 (PMC11646773; doi:10.3389/fped.2024.1452851)
Supplement: Supplementary file 1 [file Datasheet1.docx]

Supplementary Material

**Supplementary File 1.** Positions of the reflective markers from adopted Helen Hayes marker set. Front (A) and back (B) view.


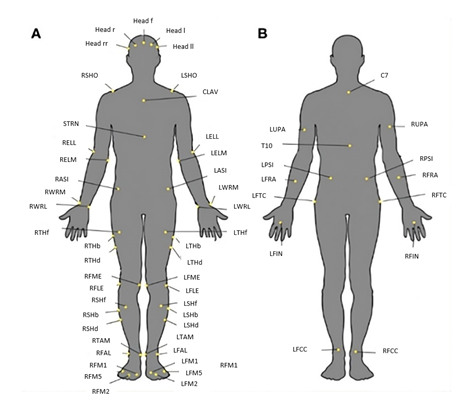


**Supplementary File 2. H1 - Descriptive statistics of all variables comparing children with and without ADHD.** Estimated marginal means, estimated marginal standard error as well as upper and lower bounds of the estimated marginal 95% confidence intervals are shown. Lower values represent better values for all motor tests, the Stroop test, and both symptom parameters. Higher values represent better values for d2-test parameters. Refer to Figures 2 and 3 for graphic representation.

| Test | no ADHD | | | | ADHD | | | |
| --- | --- | --- | --- | --- | --- | --- | --- | --- |
|  |  |  | em 95% CI | |  |  | em 95% CI | |
| motor performance | emMean | emSE | lower | upper | emMean | emSE | lower | upper |
| balance target error [cm] | 0.67 | 7.55 | 0.82 | 0.57 | 0.93 | 14.87 | 1.06 | 0.83 |
| precision jumps target error [cm] | 3.97 | 39.83 | 4.94 | 3.32 | 5.43 | 51.96 | 6.84 | 4.50 |
| One-leg stand target error [cm] | 1.08 | 14.49 | 1.27 | 0.94 | 1.26 | 28.22 | 1.38 | 1.15 |
| cognitive performance |  |  |  |  |  |  |  |  |
| d2 completed targets [number] | 119.9 | 5.5 | 108.7 | 131.0 | 100.7 | 3.1 | 94.4 | 107.0 |
| d2 concentration capacity [score] | 110.8 | 6.3 | 98.0 | 123.6 | 89.3 | 3.3 | 82.7 | 95.9 |
| Stroop time [sec] | 105.5 | 26.5 | 50.6 | 160.5 | 162.0 | 17.3 | 126.2 | 197.9 |
| symptom level |  |  |  |  |  |  |  |  |
| attention deficit [score] | 0.34 | 0.18 | -0.03 | 0.7 | 1.97 | 0.10 | 1.78 | 2.17 |
| hyperactivity [score] | 0.13 | 0.22 | -0.31 | 0.57 | 1.42 | 0.11 | 1.20 | 1.64 |

Supplementary File 3. Results of the linear model fit comparing intervention and control group for unsignificant motoric items. Effect sizes are interpreted after Cohen's rule of thumb. Asterisks denote the corresponding level of significance: *: p<.05, **: p<.01, ***: p<.001.

|  | **Term** | ***F*** | ***p*** | ***η_p_^2^*** | **effect size** |
| --- | --- | --- | --- | --- | --- |
| **Precision jumps** | Time | 0.18 | 0.67 | 0.0003 | very small |
|  | Skating | 0.23 | 0.63 | 0.0003 | very small |
|  | Cov. Age | 0.01 | 0.93 | 0.00001 | very small |
|  | Cov. Medication | 0.15 | 0.7 | 0.0002 | very small |
|  | Time × Skating | 0.04 | 0.85 | 0.0001 | very small |
| **One-leg stand** | Time | 0.36 | 0.55 | 0.002 | very small |
|  | Skating | 0.02 | 0.9 | 0.0001 | very small |
|  | Cov. Age | 1.58 | 0.21 | 0.01 | small |
|  | Cov. Medication | 2.53 | 0.11 | 0.01 | small |
|  | Time × Skating | 0.02 | 0.89 | 0.0001 | very small |

Supplementary File 4. H2 - Descriptive statistics of all variables comparing results of ADHD-affected children before and after the skateboarding intervention. Estimated marginal means, estimated marginal standard error as well as upper and lower bounds of the estimated marginal 95% confidence intervals are shown. Lower values represent better values for all motor tests, the Stroop test, and both symptom parameters. Higher values represent better values for d2-test parameters. Refer to Figures 4, 5, and 6 for graphic representation.

| Test | waitlist controls | | skateboard group | |
| --- | --- | --- | --- | --- |
|  | pre | post | pre | post |
|  | emMean ± emSE | | | |
| motor performance |  |  |  |  |
| balance target error [cm] | 1.07 ± 12.03 | 0.86 ± 14.77 | 0.99 ± 13.56 | 0.72 ± 18.2 |
| precision jumps target error [cm] | 5.41 ± 57.28 | 5.56 ± 65.94 | 5.19 ± 53.1 | 5.26 ± 68.32 |
| one leg stand target error [cm] | 1.21 ± 15.42 | 1.17 ± 14.05 | 1.22 ± 19.89 | 1.17 ± 18.78 |
| cognitive performance |  |  |  |  |
| d2 completed targets [number] | 116.3 ± 5.7 | 128.4 ± 5.3 | 103.4 ± 4.4 | 121.6 ± 4.2 |
| d2 concentration capacity [score] | 102.8 ± 6.1 | 117 ± 5.8 | 90.7 ± 4.5 | 108.9 ± 4.6 |
| Stroop time [sec] | 119.9 ± 20.9 | 94.7 ± 15.6 | 162.9 ± 17.7 | 127.6 ± 9.9 |
| symptom level |  |  |  |  |
| attention deficit [score] | 2.14 ± 0.17 | 1.82 ± 0.18 | 2 ± 0.13 | 1.61 ± 0.14 |
| hyperactivity [score] | 1.54 ± 0.19 | 1.3 ± 0.19 | 1.35 ± 0.14 | 1.09 ± 0.14 |

Supplementary File 5. Standard (not partial) correlation analysis between the investigated variables in terms of Spearman’s rho. Asterisks denote the corresponding level of significance: *: p<.05, **: p<.01, ***: p<.001.

|  | |  |  | | **Spearman's rho** | | **p** | |  |
| --- | --- | --- | --- | --- | --- | --- | --- | --- | --- |
| **Attention Deficit** |  | **-** |  | **Hyperactivity** |  | **0.715** | ******* | **< .001** |  |
| **Attention Deficit** |  | **-** |  | **D2_Concentration** |  | **-0.178** | ***** | **0.048** |  |
| Attention Deficit |  | - |  | D2_Completed |  | -0.131 |  | 0.146 |  |
| Attention Deficit |  | - |  | Stroop |  | 0.030 |  | 0.788 |  |
| Attention Deficit |  | - |  | Balance Target Error |  | 0.091 |  | 0.325 |  |
| Attention Deficit |  | - |  | One-leg stand Target Error |  | -0.076 |  | 0.403 |  |
| **Attention Deficit** |  | **-** |  | **Jump Target Error** |  | **0.267** | ***** | **0.015** |  |
| Hyperactivity |  | - |  | D2_Concentration |  | -0.128 |  | 0.157 |  |
| Hyperactivity |  | - |  | D2_Completed |  | -0.118 |  | 0.190 |  |
| Hyperactivity |  | - |  | Stroop |  | 0.006 |  | 0.958 |  |
| Hyperactivity |  | - |  | Jump Target Error |  | 0.141 |  | 0.124 |  |
| Hyperactivity |  | - |  | One-leg stand Target Error |  | -0.080 |  | 0.375 |  |
| **Hyperactivity** |  | **-** |  | **Jump Target Error** |  | **0.330** | ****** | **0.002** |  |
| **D2_Concentration** |  | **-** |  | **D2_Completed** |  | **0.936** | ******* | **< .001** |  |
| **D2_Concentration** |  | **-** |  | **Stroop** |  | **-0.615** | ******* | **< .001** |  |
| **D2_Concentration** |  | **-** |  | **Balance Target Error** |  | **-0.187** | ***** | **0.039** |  |
| **D2_Concentration** |  | **-** |  | **One-leg stand Target Error** |  | **-0.352** | ******* | **< .001** |  |
| D2_Concentration |  | - |  | Jump Target Error |  | -0.005 |  | 0.961 |  |
| **D2_Completed** |  | **-** |  | **Stroop** |  | **-0.637** | ******* | **< .001** |  |
| **D2_Completed** |  | **-** |  | **Balance Target Error** |  | **-0.209** | ***** | **0.020** |  |
| **D2_Completed** |  | **-** |  | **One-leg stand Target Error** |  | **-0.318** | ******* | **< .001** |  |
| D2_Completed |  | - |  | Jump Target Error |  | 0.096 |  | 0.385 |  |
| Stroop |  | - |  | Balance Target Error |  | 0.102 |  | 0.367 |  |
| **Stroop** |  | **-** |  | **One-leg stand Target Error** |  | **0.450** | ******* | **< .001** |  |
| Stroop |  | - |  | Jump Target Error |  | -0.186 |  | 0.117 |  |
| **Balance Target Error** |  | **-** |  | **One-leg stand Target Error** |  | **0.251** | ****** | **0.005** |  |
| Balance Target Error |  | - |  | Jump Target Error |  | 0.076 |  | 0.494 |  |
| One-leg stand Target Error |  | - |  | Jump Target Error |  | -0.095 |  | 0.392 |  |

Supplementary File 6. Heatmap graphically representing the results of the standard (not partial) correlation analysis in supplementary table 7. Darker fields correspond to stronger correlation, bluish and reddish fields correspond to positive and negative correlation, respectively. Correlation is measured in terms of Spearman’s rho. Asterisks denote the corresponding level of significance: *: p<.05, **: p<.01, ***: p<.001.

*
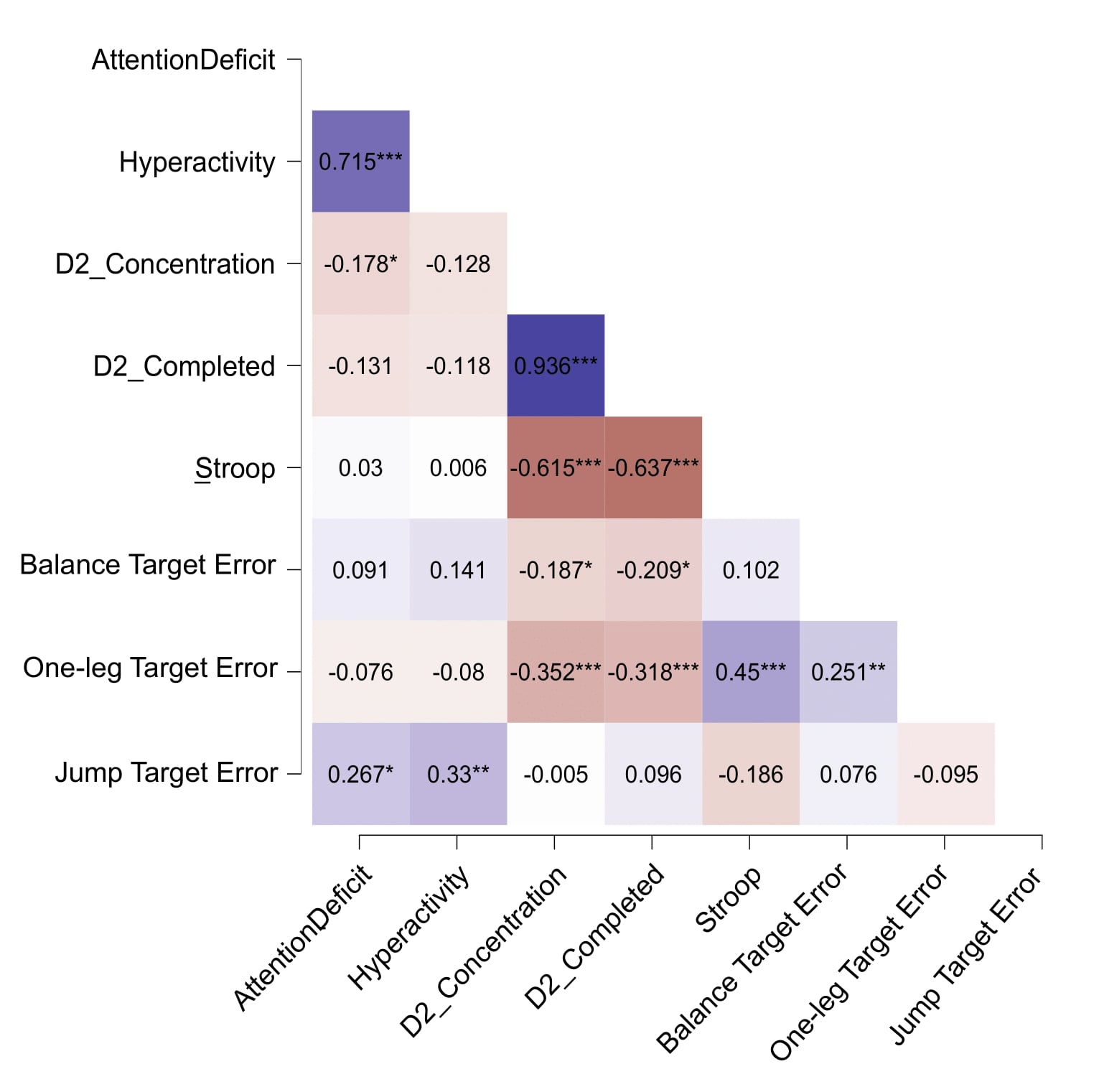
*

Supplementary File 7. Partial correlation analysis between all investigated variables, with the control variables ‘Age’, ‘ADHD’, ‘Skating’, and ‘Intervention’. Correlation is measured in terms of Spearman’s rho. Asterisks denote the corresponding level of significance: *: p<.05, **: p<.01, ***: p<.001. For a standard (not partial) correlation analysis see supplementary File 5.

|  | |  | |  | | **Spearman's rho** | | **p** | |
| --- | --- | --- | --- | --- | --- | --- | --- | --- | --- |
| **Attention Deficit** |  | **-** |  | **Hyperactivity** |  | **0.545** | ******* | **< .001** |  |
| Attention Deficit |  | - |  | D2_Concentration |  | -0.064 |  | 0.490 |  |
| Attention Deficit |  | - |  | D2_Completed |  | -0.042 |  | 0.650 |  |
| Attention Deficit |  | - |  | Stroop |  | 0.064 |  | 0.579 |  |
| Attention Deficit |  | - |  | Balance Target Error |  | -0.053 |  | 0.575 |  |
| Attention Deficit |  | - |  | One-leg stand Target Error |  | -0.063 |  | 0.492 |  |
| Attention Deficit |  | - |  | Jump Target Error |  | -0.043 |  | 0.707 |  |
| Hyperactivity |  | - |  | D2_Concentration |  | 0.041 |  | 0.660 |  |
| Hyperactivity |  | - |  | D2_Completed |  | 0.015 |  | 0.872 |  |
| Hyperactivity |  | - |  | Stroop |  | 0.057 |  | 0.621 |  |
| Hyperactivity |  | - |  | Balance Target Error |  | 0.007 |  | 0.938 |  |
| Hyperactivity |  | - |  | One-leg stand Target Error |  | -0.077 |  | 0.405 |  |
| Hyperactivity |  | - |  | Jump Target Error |  | 0.058 |  | 0.615 |  |
| **D2_Concentration** |  | **-** |  | **D2_Completed** |  | **0.891** | ******* | **< .001** |  |
| **D2_Concentration** |  | **-** |  | **Stroop** |  | **-0.462** | ******* | **< .001** |  |
| D2_Concentration |  | - |  | Balance Target Error |  | -0.137 |  | 0.137 |  |
| **D2_Concentration** |  | **-** |  | **One-leg stand Target Error** |  | **-0.354** | ******* | **< .001** |  |
| D2_Concentration |  | - |  | Jump Target Error |  | -0.026 |  | 0.822 |  |
| **D2_Completed** |  | **-** |  | **Stroop** |  | **-0.477** | ******* | **< .001** |  |
| D2_Completed |  | - |  | Balance Target Error |  | -0.175 |  | 0.056 |  |
| **D2_Completed** |  | **-** |  | **One-leg stand Target Error** |  | **-0.306** | ******* | **< .001** |  |
| D2_Completed |  | - |  | Jump Target Error |  | 0.082 |  | 0.472 |  |
| Stroop |  | - |  | Balance Target Error |  | 0.013 |  | 0.910 |  |
| **Stroop** |  | **-** |  | **One-leg stand Target Error** |  | **0.406** | ******* | **< .001** |  |
| Stroop |  | - |  | Jump Target Error |  | -0.047 |  | 0.704 |  |
| **Balance Target Error** |  | **-** |  | **One-leg stand Target Error** |  | **0.242** | ****** | **0.008** |  |
| Balance Target Error |  | - |  | Jump Target Error |  | -0.004 |  | 0.970 |  |
| One-leg stand Target Error |  | - |  | Jump Target Error |  | -0.053 |  | 0.641 |  |
|  | | | | | | | | | |
| *Note.*  Standard error of effect size (Fisher's z) is currently unavailable for non-parametric partial correlations. | | | | | | | | | |
